# Membrane Particles Derived From Adipose Tissue Mesenchymal Stromal Cells Improve Endothelial Cell Barrier Integrity

**DOI:** 10.3389/fimmu.2021.650522

**Published:** 2021-04-07

**Authors:** Ana Merino, Marta Sablik, Sander S. Korevaar, Carmen López-Iglesias, Maitane Ortiz-Virumbrales, Carla C. Baan, Eleuterio Lombardo, Martin J. Hoogduijn

**Affiliations:** ^1^ Nephrology and Transplantation, Department of Internal Medicine, Erasmus Medical Center, Rotterdam, Netherlands; ^2^ Microscopy CORE Lab, Maastricht Multimodal Molecular Imaging Institute, FHML Maastricht University, Maastricht, Netherlands; ^3^ Takeda Madrid, Cell Therapy Technology Center, Madrid, Spain

**Keywords:** membrane particles, nanovesicles, mesenchymal stromal cells, endothelial cells, regeneration, immune cell interaction

## Abstract

Proinflammatory stimuli lead to endothelial injury, which results in pathologies such as cardiovascular diseases, autoimmune diseases, and contributes to alloimmune responses after organ transplantation. Both mesenchymal stromal cells (MSC) and the extracellular vesicles (EV) released by them are widely studied as regenerative therapy for the endothelium. However, for therapeutic application, the manipulation of living MSC and large-scale production of EV are major challenges. Membrane particles (MP) generated from MSC may be an alternative to the use of whole MSC or EV. MP are nanovesicles artificially generated from the membranes of MSC and possess some of the therapeutic properties of MSC. In the present study we investigated whether MP conserve the beneficial MSC effects on endothelial cell repair processes under inflammatory conditions. MP were generated by hypotonic shock and extrusion of MSC membranes. The average size of MP was 120 nm, and they showed a spherical shape. The effects of two ratios of MP (50,000; 100,000 MP per target cell) on human umbilical vein endothelial cells (HUVEC) were tested in a model of inflammation induced by TNFα. Confocal microscopy and flow cytometry showed that within 24 hours >90% of HUVEC had taken up MP. Moreover, MP ended up in the lysosomes of the HUVEC. In a co-culture system of monocytes and TNFα activated HUVEC, MP did not affect monocyte adherence to HUVEC, but reduced the transmigration of monocytes across the endothelial layer from 138 ± 61 monocytes per microscopic field in TNFα activated HUVEC to 61 ± 45 monocytes. TNFα stimulation induced a 2-fold increase in the permeability of the HUVEC monolayer measured by the translocation of FITC-dextran to the lower compartment of a transwell system. At a dose of 1:100,000 MP significantly decreased endothelial permeability (1.5-fold) respect to TNFα Stimulated HUVEC. Finally, MP enhanced the angiogenic potential of HUVEC in an *in vitro* Matrigel assay by stimulating the formation of angiogenic structures, such as percentage of covered area, total tube length, total branching points, total loops. In conclusion, MP show regenerative effects on endothelial cells, opening a new avenue for treatment of vascular diseases where inflammatory processes damage the endothelium.

## Introduction

The endothelium forms an interactive barrier between the circulatory system and the tissues in the body. It plays a pivotal role in the regulation of vascular permeability, hemostasis, and immunological processes (1). Alterations of endothelial cells (EC) play a central role in the pathogenesis of a broad spectrum of the most dreadful of human diseases, such as atherosclerosis (2), stroke (3), heart disease (4), diabetes (5), allograft rejection (6), and chronic kidney failure (7). Inflammatory mediators cause overexpression of cell adhesion molecules (CAM) on EC and together with the secretion of cytokines this permits the attraction and adhesion of circulating immune cells to the endothelium, and consequently, the transmigration of leukocytes into inflammation sites (8). Therapies that protect the endothelium from stress and immune factors or enhance the repair processes may be capable of curing or preventing diseases where the endothelium has a key role.

Mesenchymal stromal cells (MSC) represent such therapy as they have immunomodulatory and regenerative capacities and are known to deliver endothelial protective signals (9). The endothelial protective effects of MSC are due to their anti-inflammatory and repair properties that have shown substantial therapeutic promise in preclinical models, such as for instance in atherosclerosis (10). Moreover, MSC hold great promise for revascularization of tissues as they secrete pro-angiogenic and anti-apoptotic factors in large amounts (11).

The translation of the endothelial protective and reparative effects of MSC found in the *in vitro* setting to an effective therapy is hampered by the poor biodistribution of infused MSC after intravenous administration. It is demonstrated that after intravenous infusion, MSC get trapped in the lungs and have a short survival time (12, 13). This implies that MSC do not reach sites of injury and cannot interact locally with injured tissue. Viable MSC may secrete cytokines and growth factors in the circulation and target distant cells *via* this route, but recent work demonstrated that inactivated MSC, which lost their capacity to secrete factors, maintain their immunomodulatory capacity in an animal model (14), suggesting that cell membrane dependent interactions with target cells play a role in the immune regulatory effects of MSC. Furthermore, MSC-conditioned media have shown to possess similar regenerative properties as MSC on tissue damage and contribute to the modulation of inflammation (15). Conditioned medium is composed of growth factors, cytokines, and extracellular vesicles (EV). EV are spherical membrane fragments heterogeneous in size and composition that carry and transfer proteins, lipids, and RNA from the source cells to resident cells in damaged tissue (16). MSC-derived EV have shown therapeutic effects in several diseases’ models including CVD (17) and acute kidney injury (18). Despite EV may be a promising alternative cell-free therapy, clinical translation is hindered by the lack of suitable and scalable technologies for the generation and purification of extracellular vesicles (19, 20). Thus, novel methods are needed to make pharmaceutically controllable and homogeneous membrane vesicles for targeting injured tissues.

We previously reported on the generation of large amounts of membrane particles (MP) from human adipose tissue MSC (AT-MSC) (21). The size of these man-made MP was with on average 120 nm, like naturally occurring EV, and electron microscopy showed they have a spherical shape. MP were shown to be able to modulate immune cells, thereby showing a great potential as a novel cell-free immune therapy, and a good alternative to EV therapy as MP can be produced in large amounts, highly purified, in an easy and economic process.

In the present study, we have investigated the potential therapeutic effects of MP derived from AT-MSC on the barrier integrity of inflamed endothelial cells using a model of TNFα treated human umbilical vein endothelial cells (HUVEC). We further explored whether MP could enhance the angiogenic ability of HUVEC in an inflammatory environment.

## Material And Methods

### Ethics Statement and Human Tissue Samples

Human MSC were isolated from subcutaneous adipose tissue from healthy kidney donors that became available during kidney donation procedures. The tissues were collected after obtaining written informed consent, as approved by the Medical Ethical Committee of the Erasmus University Medical Centre Rotterdam (protocol no. MEC-2006-190).

### Isolation and Culture of MSC From Adipose Tissue

AT-MSC were isolated from subcutaneous adipose tissue of five healthy donors (2 females/3 males). The age of the donors was between 34-58 years old. The tissue was mechanically disrupted and enzymatically digested with 0.5 mg/ml collagenase type IV (Sigma-Aldrich, St. Louis, MO) in RPMI for 30 min at 37°C under continuous shaking. Thereafter, the cells were resuspended in MEM-*α* with 10% fetal bovine serum (FBS; Lonza, Verviers, Belgium), 2 mM L-glutamine and 1% P/S, filtered through a 100 µm cell strainer, and transferred to 175 cm^2^ culture flasks (Greiner Bio-one, Essen, Germany). At 90% confluence AT-MSC (passage 2-6) were collected to generate MP. The phenotypic characterization of AT-MSC was performed by flow cytometry using FACSCANTO-II with FACSDIVA Software (BD Biosciences, San Jose, CA). AT-MSC were incubated with mouse-anti-human monoclonal antibodies against CD13-PE-Cy7; HLA-DR-PERCP; HLA-ABC-APC; CD31-FITC; CD73-PE; PD-L1-PE (all BD Biosciences); CD90-APC and CD105-FITC (R&D Systems, Abingdon, UK). All the antibodies were incubated with the cells for 30 min, at room temperature in the absence of light.

### Culture of Human Umbilical Vein Endothelial Cells

First-passage cryopreserved HUVEC from pooled donors were obtained from Promocell (Promocell, Germany). HUVEC were grown in 75 cm^2^ flasks at 37 °C, 5% CO_2_ with endothelial cell basal medium (EBM, Cambrex Bio Science Walkersville, Inc., Walkersville, MD, USA), endothelial cell growth medium supplements (EGM, Cambrex Bio Science), 10% FBS, 100 IU/mL penicillin, and 100 mg/mL streptomycin. At 90% confluence, HUVEC were dissociated by 0.05% trypsin-EDTA (Life Technologies, Bleiswijk, The Netherlands). To establish an endothelial cell model of inflammation, HUVEC were incubated with TNFα (25ng/ml) for 4h or 24h depending on the assay. All HUVEC used in the experiments were between passage 2-7. During these passages, HUVEC conserved their morphology, phenotype, and proliferation rate. For the stimulation of HUVEC with TNFα, three concentrations of TNFα were tested (10, 25, 50ng/ml). All the experiments were performed with the concentration 25 ng/ml due to the difference respect to adhesion molecules and monocyte adhesion assay between Control and TNFα treated cells was enough to allow MP play a role, without inducing HUVEC apoptosis.

### Generation of Membrane Particles From AT-MSC

**Figure 1:**
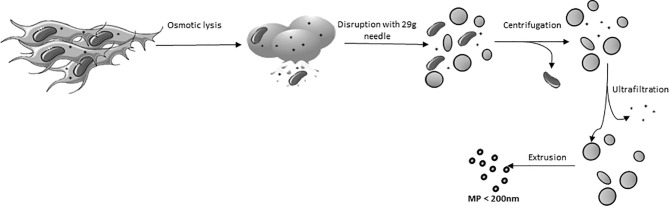
Schematic overview of the generation of Membrane particles.

AT-MSC were trypsinized and washed twice with PBS. Then, the MSC were incubated in milliQ water at 4°C to induce osmotic lysis and liberation of the cell nuclei (after about 20 min, monitored by microscope). Cell extracts were cleared of unbroken cells and nuclei by centrifugation at 2,000 x *g* for 20 min. The obtained supernatant was transferred to Amicon Ultra-15 filter tubes (100 kDa pore size) and concentrated by centrifugation at 4,000 x *g* at 4°C. The concentrated pellet consisted of crude membrane and was diluted in 0.2 µm filtered PBS. A population of MP, homogeneous in size was obtained by extruding the plasma membranes 3 times through polycarbonate membrane filters (Merck, KGaA, Darmstadt, Germany), first with a pore diameter of 800 nm, secondly with a 400 nm and last with a 200 nm pore size filter. The extrusion process was performed using LiposoFast LF-50 (AVESTIN Europe, Mannheim, Germany) at 20 psi (**Figure 1**). All procedures were performed on ice.

### Analysis of Adhesion Markers on HUVEC

HUVEC were incubated with TNFα (25ng/ml) and two ratios of MP (1:50,000, 1:100,000 HUVEC : MP) during 24h. Then, the cells were trypsinized and washed with FACS Flow (BD Biosciences, San Jose, CA). The immunophenotypic characterization of the activation state of endothelial cells was done by incubating HUVEC with mouse-anti-human monoclonal antibodies against CD54-APC, CD106-BV421, CD62e-PE, CD31-FITC, VEGFR2-PE, CD105-FITC and TIE2-Alex647 (all BD Biosciences). All the antibodies were incubated with the cells for 30 min, at room temperature in the absence of light. After two washes with FACS Flow, flow cytometric analysis was performed using FACSCANTO-II with FACSDIVA Software (BD Biosciences).

### Characterization of MP Size and Concentration by Nanoparticle Tracking Analysis

Analysis of absolute size distribution and concentration of MP was performed using NanoSight NS300 (NanoSight Ltd.). With NTA, particles are automatically tracked and sized based on Brownian motion and the diffusion coefficient. The NTA measurement conditions were: detection threshold 3 (determined with a protein solution), three measurements per sample (30 s/measurement), temperature 23.61 ± 0.8°C; viscosity 0.92 ± 0.02 cP, frames per second 25. Each video was analyzed to give the mean, mode, median and estimated concentration for each particle size. The samples were diluted in 0.2µm filtered PBS, to obtain a measurable concentration of particles (1 x 10^8^ particles/ml) in accordance with the manufacturer’s recommendations.

### Cryo-Transmission Electron Microscopy

The preparations of MP were visualized by the Cryo-TEM method. A thin aqueous film was formed by applying a 3µl droplet of MP suspension to a specimen bare EM grid. Glow-discharged holey carbon grids were used. After the application of the suspension the grid was blotted against filter paper, leaving a thin sample film spanning the grid holes. These films were vitrified by plunging the grid into ethane, which was kept at its melting point by liquid nitrogen, using a Vitrobot (Thermo Fisher Scientific Company, Eindhoven, The Netherlands). The vitreous sample films were transferred to a Tecnai Arctica microscope (Thermo Fisher Scientific, Eindhoven, The Netherlands). Images were taken at 200 Kv with a field emission gun using a Falcon III (Thermo Fisher Scientific) direct electron detector.

### Extraction and Identification of DNA/RNA From MP

To examine whether DNA and RNA are present in MP, a High Pure RNA Isolation Kit (Roche Applied Science, Penzberg, Germany) was used to extract DNA/RNA from MP samples following the manufacturer’s instructions. After the isolation of the RNA/DNA, the samples were treated with DNase I to quantify the concentration of RNA, whereas for the collection of both DNA and RNA, DNase I treatment was omitted. The concentration and purity of DNA+RNA and RNA in the samples was assessed spectrophotometrically using a NanoDrop ND-1000 (Thermo Fisher Scientific, Bleiswijk, The Netherlands). The quality of the RNA was assessed by assigning an RNA integrity number (RIN) using an Agilent 2100 Bioanalyzer (Agilent Technologies, Santa Clara, CA, USA).

### Quantitative RT-PCR Analysis

MP were stored at −80°C. Total RNA was isolated, and 500 ng used for complementary DNA (cDNA) synthesis. Gene expression was determined by Quantitative Real-Time PCR (qPCR) using the TaqMan Universal PCR Master Mix (Life Technologies ThermoFisher scientific), and the assay-on-demand primer/probes for Thermo Fisher GAPDH (Hs99999905.m1); CD90 (Hs00264235_s1), Vascular endothelial growth factor A (VEGF-A: Hs00173626.m1), Angiopoietin 1 (Hs01586213.m1), IL-8 (Hs00174114.m1). For PCR, cDNA synthesized from 25 ng total RNA was used to perform each amplification.

### Assessment of MP Toxicity on HUVEC: Apoptosis Assay

HUVEC were seeded at a density of 2x10^5^/well in 12-well plates. Then, unstimulated and TNFα (25ng/ml) stimulated HUVEC were cultured with 2 ratios of MP (HUVEC : MP 1:50,000, 1:100,000) during 24h and 48h. Cell viability was assessed using an Annexin V staining kit (BD Biosciences) according to the manufacturer’s recommendations. Briefly, after the incubation time with MP, cells were harvested, washed in PBS, and resuspended in a binding buffer that contained 5 μl Annexin V antibody and 5 μl 7-AAD. Samples were measured by FACSCanto II (BD Biosciences).

### Uptake of MP by HUVEC

AT-MSC were labeled with red fluorescent PKH-26 dye, which intercalates into lipid bilayers, according to the manufacturer’s instructions (Sigma-Aldrich), enabling the generation of fluorescent MP (PKH-MP). HUVEC were plated at a density of 2x10^5^ cells/well on a 12 well plate, treated with/without TNFα (25ng/mL). Two ratios of PKH-MP, (1:50,000 and 1:100,000) were added to the cultures for 4h and 24h and the uptake of MP by HUVEC was quantified by flow cytometry. The data were analyzed using Kaluza Software (Beckman Coulter).

For confocal microscopy analysis, cell membranes of HUVEC were labeled with PKH-67, the nuclei with 10µM Hoechst 33342, and the lysosomes with a LysoSensor dye (Invitrogen Molecular Probes), which changes to yellow fluorescence in acidic environments. PKH-MP uptake by HUVEC was imaged by a Leica TCS SP5 confocal microscope (Leica Microsystems B.V., Science Park Eindhoven, Netherlands), equipped with Leica Application Suite – Advanced Fluorescence (LAS AF) software, DPSS 561 nm lasers, using a 40 X (1.4 NA oil) objective. Microscopic images were processed using ImageJ 1.48 (National Institutes of Health, Washington, USA).

### Monocyte Adhesion Assay

HUVEC were seeded at 0.5x10^6^ cells/well in a 12 well plate and TNFα added at 25ng/mL for 24h in combination with MP at a ratio of 1:50,000. Peripheral blood mononuclear cells (PBMC) were isolated from a buffy coat of healthy individuals. Monocytes were purified from the buffy coat using auto-MACS Pro by negative-selection (Miltenyi Biotec, Germany). The purified monocytes were labeled with 1 µM of CFSE and kept in suspension (1x10^6^ cells/mL) in culture medium consisting of RPMI 1640 medium (Life Technologies), supplemented with 10% FBS, 100 IU/mL penicillin and 100 mg/mL streptomycin. Monocyte purity was checked using flow cytometry after staining with mouse-anti-human monoclonal antibody against CD14 (BD Biosciences) for 20 min at room temperature. CFSE-labeled monocytes (1x10^5^ per condition) were added to the stimulated HUVEC and incubated for 1h at 37 °C, 5% CO_2_. The incubation time of 1h was determined as the time where monocytes in suspension were not anymore observed in the TNFα condition (positive control). After a thorough wash with EBM, the cultures were photographed with a Leica TCS SP5 confocal microscope. Microscopic images were processed using ImageJ 1.48. Stained cells were counted in five randomly selected areas using bright field microscopy (× 20).

### Transwell Cell Transmigration Assay

HUVEC (1x10^5^ cells/transwell) were plated in Transwell^®^-24 well inserts (Costar, Corning Inc.), consisting of polycarbonate filters (8 μm pore size; 0.33 cm^2^ area), and grown to confluence for 24h. HUVEC were then treated with 25 ng/mL TNFα and 50,000 MP per HUVEC for 24h. Then, the supernatant was discarded and the transwells were transferred to a new well containing 500µL of 50 ng/mL Monocyte Chemoattractant Protein-1 (MCP-1, Invitrogen Molecular Probes) in the lower well. Monocytes were isolated, labeled with PKH-26, and plated in the transwell at a ratio of 2:1 (monocyte:HUVEC). Following 2 hours of incubation at 37 °C and 5% CO_2_, the supernatant of the transwells was carefully removed together with the non-adhering monocytes. The adherent cells were washed twice with PBS and stained with 10µM Hoechst 33342 for 10 min at 37 °C, 5% CO_2_. The inserts were then washed twice with PBS and fixed with 4% formaldehyde dissolved in PBS for 15 min at room temperature. Monocytes that migrated through both the HUVEC monolayer and polycarbonate membrane and adhered to the bottom side of the transwell membrane were visualized by Z-stacks analysis using a Leica TCS SP5 confocal microscope. The number of transmigrated monocytes was determined by counting the number of PKH-26 fluorescent monocytes present in 5 randomly selected fields of view per sample *via* ImageJ 1.48.

### Transwell Permeability Assay

To analyze the endothelial cell barrier integrity 50,000 HUVEC were grown on a transwell insert pre-coated with fibronectin (polystyrene, 0.4 um pore size; Greiner Bio-one, The Netherlands) until confluency. The monolayers of HUVEC were then treated with 25 ng/mL TNFα and two ratios of MP (50,000 and 100,000 MP : HUVEC) for 24h. After the incubation time, the supernatant was removed and FITC-dextran (1mg/ml; 70kDa; Bio-connect, The Netherlands) was added to the transwells. After 2h, the FITC-dextran translocated to the lower compartment of the transwell was measured in a microplate reader at excitation/emission wavelength of 490/520nm. As a positive control a transwell without cells was used. By normalizing the fluorescence signals of the treatment group to the control group a measure of endothelial layer leakiness was obtained.

### Angiogenesis Assay/Tube Formation Assay

A confluent monolayer of HUVEC was treated with MP at a ratio of 1:50,000 and treated with/without TNFα (25ng/ml) for 24h. After the incubation time, HUVEC were collected by trypsinization and seeded on 50µl polymerized Matrigel (Geltrex, ThermoFisher, USA). The major components of Geltrex™ matrix include laminin, collagen IV, entactin, and heparin sulfate proteoglycans. The protein concentration is 15mg/ml. Each condition was plated in duplicate. After 18h, tube formation was observed and photographed using an inverted light microscope equipped with a digital camera. The percentage of covered area (percentage of tubular structures in the whole area of the image), total tube length (complete length in pixel of the tubular structure), total branching points (a branching point is part of the skeleton where three or more tubes converge), and the number of loop areas (a loop is an area enclosed by tubular structures) were measured by WIMASIS (Onimagin Technologies SCA, Córdoba, Spain).

### Sample Size and Statistical Analysis

In the experiments MP from 5 donors were used in duplicate. For the apoptosis, expression of adhesion markers and monocyte adhesion assays, 5 independent experiments were performed where MP from 2 different donors were tested in each experiment. For the monocyte migration assay, barrier integrity and angiogenesis assays, 3 independent experiments were performed where MP from 3 different donors were tested in each experiment. Data were analyzed for normal distribution by Kolmogorov-Smirnov test, and after that T-Test was used to determine the significance between the groups using GraphPad Prism 5 software. P < 0.05 was considered significant.

## Results

### Morphology and Size Distribution of MP Generated From AT-MSC

**Figure 2:**
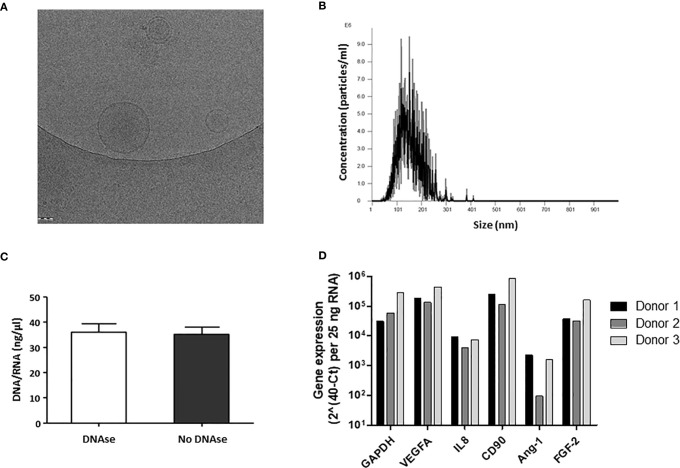
Characterization of physical properties and DNA/RNA composition of membrane particles from MSC. **(A)** Cryo-electron microscopy images of MP. MP show a spherical shape and a discernible lipid bilayer. **(B)** A representative profile of the nanoparticle tracking analysis (NTA) of MP. A graph was generated which plots the distribution in size of the MP against the concentration of MP per ml. **(C)** RNA/DNA concentration (ng/µl) in MP samples before and after DNAse treatment. The error bars represent standard deviation of the mean (SD). **(D)** Relative gene expression of RNA present in MP samples from three different MSC donors.

MP were generated from culture-expanded AT-MSC and characterized by cryo-electron microscopy and NTA to determine their shape, concentration, and size distribution. Cryo-electron microscopy showed that MP have a spherical shape and a discernible lipid bilayer (**Figure 2A**). Some MP were found encapsulated inside larger MP. The size range of MP was between 32 and 345 nm, with an average peak size frequency of 126.5± 22.4 nm. The frequency of particles larger than 200nm (cut-off pore size) was lower than 0.5 ± 0.3% (**Figure 2B**).

### Presence of RNA in MP

To examine whether MP preparations contained DNA and RNA, DNA and RNA concentrations were determined by Nanodrop. MP preparations contained 35.2 ± 3.9 ng/ul DNA/RNA (**Figure 2C**). After DNase treatment, the concentration of DNA/RNA did not change (**Figure 2C**), suggesting MP contain RNA, but no DNA. To determine whether the RNA could be detected by RT-PCR, several genes expressed by MSC were analyzed. PCR product was obtained for GAPDH, the angiogenic genes VEGFA, angiopoietin 1 and FGF-2, IL-8, and for the MSC cell surface marker CD90 (**Figure 2D**) suggesting that MP preparations contained RNA from the cell source (**Figure 2D**).

### HUVEC Internalize Membrane Particles in a Time Dependent Manner

**Figure 3:**
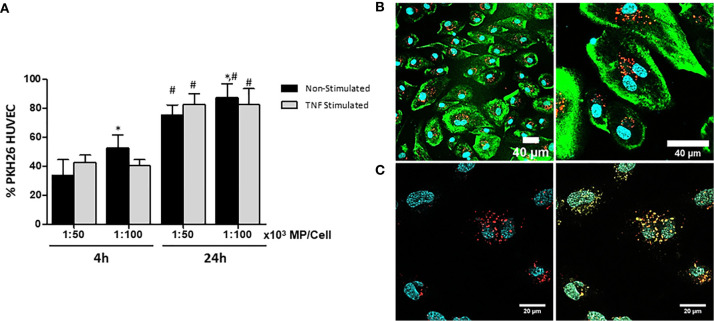
Characterization and quantification of uptake of MP by unstimulated and TNFα-stimulated HUVEC. MSC were labeled with PKH-26 before generation of MP (PKH-MP). PKH-MP were added to HUVEC (ratio 1:50,000) and incubated for 4 and 24h at 37°C. **(A)** Uptake of PKH-MP by unstimulated and TNFα-stimulated HUVEC (ratio 1:50,000 and 1:100,000) was quantified using flow cytometry. Uptake is indicated by PKH-MP positive HUVEC (PKH+ HUVEC). **(B)** Representative confocal microscopy analysis of PKH-MP uptake by HUVEC at time point 24h. Staining for PKH26-MP (red), PKH-67 cell membrane (green), and Hoechst 33342 nucleus (blue) showed that PKH-MP are internalized by HUVEC. Scale bars: 40 μm **(C)** Staining for PKH-MP (red), lysosomes (yellow) and nucleus (blue) showed that PKH-MP (ratio 1:50,000) are co-localized with lysosomes in HUVEC after 24h of incubation. Scale bars: 20 μm.

Fluorescent MP were generated by labeling the cell membranes of MSC with PKH-26 (PKH-MP). HUVEC were incubated with or without TNFα and with two ratios of PKH-MP (ratio: 1:50,000 or 1:100,000) for 4h or 24h. Non-Stimulated HUVEC showed a significant increase in the internalization of the PKH-MP with increasing MP dose and over time. However, there was not statistical difference in the internalization of PKH-MP in TNFα Stimulated HUVEC between the two tested MP doses, but there was a significant increase over time (**Figure 3A**). For the ratios 1:50,000 and 1:100,000 the percentage of Non-Stimulated HUVEC positive for PKH-MP was 75.2 ± 6.3%, and 86.8 ± 7.4% respectively after 24h of incubation, and for TNFα Stimulated HUVEC were 82.1 ± 7.1%, and 82.4 ± 11.2%. There was not statistical difference between TNFα and Non-Stimulated HUVEC.

The interaction of PKH-MP with HUVEC was visualized using confocal immunofluorescence microscopy. The analysis showed that PKH-MP were internalized and localized in the cytoplasm of HUVEC (**Figure 3B**). Subsequently, a LysoSensor staining was used to examine whether PKH-MP end up in lysosomes. The LysoSensor staining fluorescently labels endosomes and turns yellow when the pH in the endosomes is acidic, indicative for lysosomes. After incubating HUVEC with PKH-MP for 24 h, fluorescently labeled MP co-localized with lysosomes (**Figure 3C**).

### Membrane Particles Do Not Induce Apoptosis or Affect the Expression of Adhesion Molecules in HUVEC

**Figure 4:**
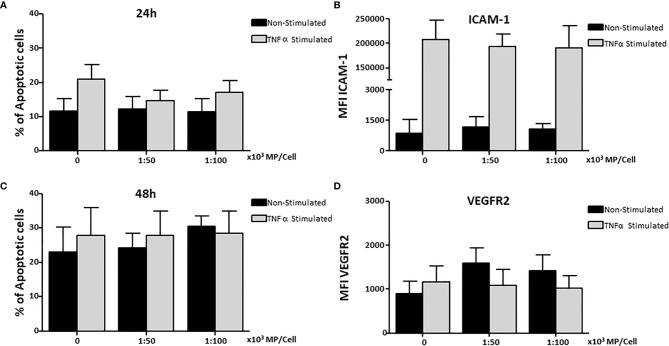
Effect of Membrane Particles on HUVEC apoptosis and adhesion molecules. HUVEC were stimulated with TNFα and treated with two concentration of MP (1:50,000, 1:100,000) and incubated at **(A)** 24h, and **(B)** 48h for the analysis of apoptosis. Surface expression of adhesion molecules on HUVEC was measured at the time point of 24h **(C)** ICAM-1, and **(D)** VEGFR2. Values are means ± SD of the mean fluorescent intensity of the receptors of 5 independent experiments each testing MP from 2 donors in each experiment.

HUVEC were stimulated with TNFα and cultured with two concentrations of MP (1:50,000 and 1:100,000) for 24h and 48h to determine whether MP induce apoptosis. No increase in apoptosis was observed in non-stimulated and TNFα stimulated HUVEC treated with MP at 24h (**Figure 4A**) or 48h (**Figure 4B**).

HUVEC were cultured with MP for 24h to determine whether MP could influence adhesion molecules expression (CD54, CD106, CD62e, CD31, CD105) and molecules involved in angiogenesis (VEGFR2, TIE-2) in non-stimulated and TNFα stimulated HUVEC. MP did not modify the expression of ICAM-1 in non-stimulated or TNFα-stimulated HUVEC (**Figure 4C**) or VEGFR2 (**Figure 4D**). In addition, no changes were observed for the rest of molecules (data not shown).

### MP Do Not Affect the Adhesion of Monocytes to HUVEC

**Figure 5:**
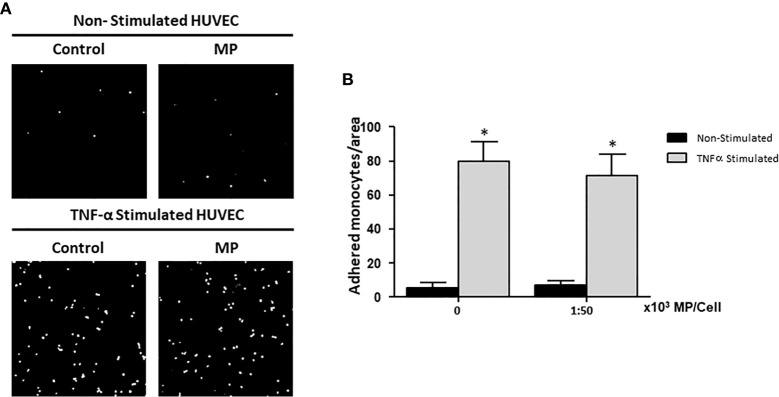
Effects of Membrane Particles on monocyte adhesion to TNFα-activated HUVEC. HUVEC were stimulated with TNFα and treated with MP at ratio 1:50,000. Subsequently, CFSE-labeled monocytes were added during 1h. **(A)** Representative fluorescent microscopy pictures show the adhered monocytes (white dots) to the HUVEC monolayer in non-stimulated and TNFα stimulated conditions. **(B)** Quantitative results of the monocyte adhesion assay analyzed by imageJ. No significance difference respect to the respective control (Non-Stimulated, and TNFα Stimulated HUVEC) was observed when MP were added *p < 0.05 compared to Non-Stimulated HUVEC.

HUVEC were treated with or without TNFα for 24h and 1:50,000 MP per HUVEC. Subsequently, the HUVEC were co-cultured for 1h with CFSE-labeled monocytes to examine the adhesion of monocytes to HUVEC. After washing away non-adherent cells, monocyte adhesion was quantified by analysis of confocal microscopy images. Digital images were captured at ×20 magnification and analyzed by ImageJ software. **Figure 5A** shows representative images of the studied groups. Activating the HUVEC using TNFα significantly increased the number of monocytes adhering to the HUVEC compared to non-stimulated HUVEC (**Figure 5B**). There was no effect of MP on the adhesion of monocytes to Non-Stimulated and TNFα Stimulated HUVEC.

### Inhibition of Monocyte Transendothelial Migration by Membrane Particles

**Figure 6:**
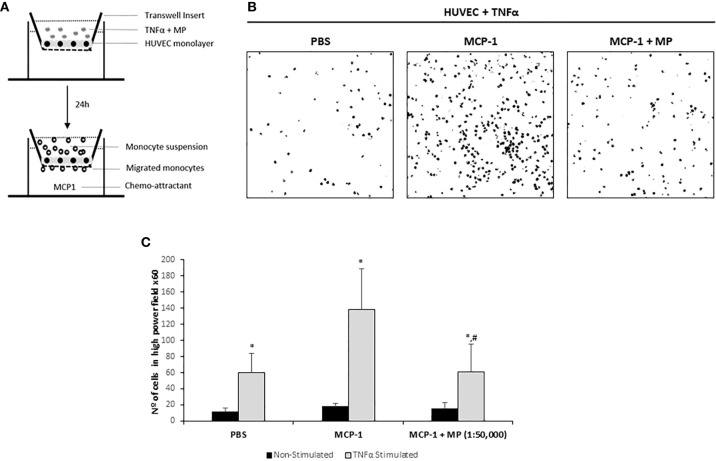
Effect of Membrane Particles on migration of monocytes through a monolayer of TNFα-activated HUVEC. **(A)** Schematic representation of the transmigration assay. HUVEC were seeded on transwell inserts until confluency. The monolayer of cells was treated with TNFα and 1:50,000 MP for 24h. Then, 1x10^5^ isolated monocytes were added during 2h with addition of the chemo-attractant MCP-1 in the bottom well. Three pictures from randomly selected areas of the transwell were taken for the quantification. **(B)** Representative confocal microscopy images of the negative control (no MCP-1), positive control (MCP-1) and the MP treated group analyzed by ImageJ. **(C)** Quantitative results of the transmigration assay. Data represent means ± SD of the number of transmigrated monocytes. *p < 0.05 respect to Non-Stimulated HUVEC. ^#^p < 0.05 respect to TNFα stimulated HUVEC non treated with MP in the MCP-1 group.

To examine the effect of MP on the transendothelial migration potential of monocytes, a HUVEC monolayer on a transwell membrane was treated with TNFα and/or MP and after 24h fluorescent monocytes were added (**Figure 6A**). Monocyte transmigration across the endothelial layer was observed at 2h. Representative confocal microscopy pictures of the assay are shown in **Figure 6B**. Addition of the chemo-attractant (MCP-1) to the lower well significantly increased the number of migrated monocytes 2.3-fold compared to PBS (PBS: 59.7 ± 24.3; MCP-1: 138.2 ± 61 migrated monocytes per microscopic field). MP were able to significantly reduce the number of monocytes that migrated through the TNFα activated HUVEC monolayer (61.3 ± 44.6 migrated monocytes per microscopic field) compared to the MCP-1 (**Figure 6C**). Non TNFα activated HUVEC treated with MP were used to examine whether MP could induce monocyte transmigration under non-inflammatory conditions. The addition of MCP-1 did not induce an increase in transmigrated monocytes (18.2 ± 3,4 migrated monocytes per microscopic field) compared to PBS (11 ± 5.2 migrated monocytes per microscopic field). The number of transmigrated monocytes in the MP treated HUVEC was similar to the Non-treated HUVEC (15.3 ± 7.1 migrated monocytes per microscopic field). The number of monocytes that migrated through the Non-Stimulated HUVEC monolayer was very low compared to TNFα-Stimulated HUVEC and the number of monocytes that adhered to the Non-Stimulated HUVEC was also very low (**Figure 6C**).

### MP Increase Endothelial Monolayer Integrity

**Figure 7:**
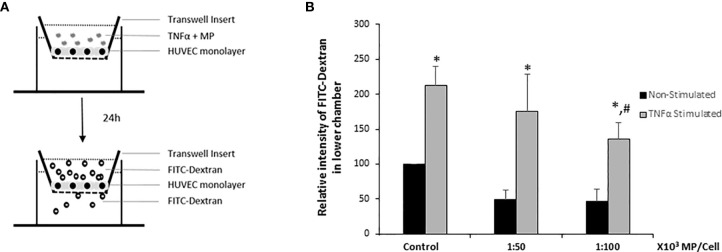
HUVEC barrier integrity. **(A)** Schematic representation of the endothelial barrier model used in the study. HUVEC were seeded on transwell inserts until confluency and then treated with TNFα and MP (1:50,000 and 1:100,000) during 24h. FITC-dextran was added during 2h, after which the fluorescence intensity in the lower chamber of the transwell system was quantified. **(B)** Quantitative results of the HUVEC barrier integrity assay. Data represent means ± SD of 3 experiments using MP from 5 different donors. *p < 0.05 compared with the respective control (Non-Stimulated HUVEC). ^#^p < 0.05 respect to TNFα stimulated HUVEC (Control).

To analyze whether MP induce a decrease in endothelial intercellular permeability, HUVEC were cultivated as tight monolayers in a transwell system and were treated or not with TNFα, and two ratios of MP (1:50,000; 1:100,000) during 24h. Thereafter, permeability was determined by measuring the passage of FITC-Dextran (molecular mass: 70 kDa) across HUVEC monolayers (**Figure 7A**). Results were normalized to the Non-Stimulated HUVEC control group. The analysis showed that both doses of MP decreased the endothelial permeability in Non-Stimulated HUVEC. TNFα stimulation induced a 2-fold increase in the permeability of the monolayer compared to the Non-Stimulated control. At a dose of 1:100,000 MP significantly decreased endothelial permeability (**Figure 7B**).

### MP Have Pro-Angiogenic Properties

**Figure 8:**
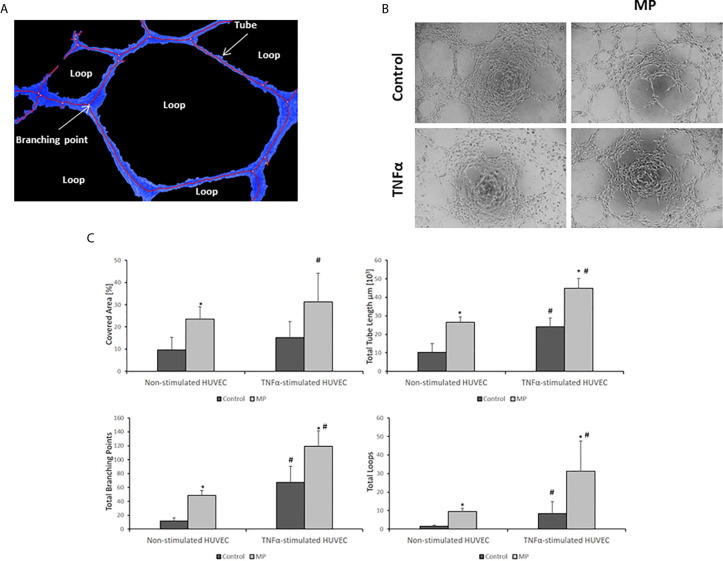
MP induce angiogenesis in Non- and TNFα Stimulated conditions. **(A)** Analysis and identification of angiogenic features. **(B)** Angiogenesis assay images of Non-Stimulated and TNFα stimulated HUVEC under MP treatment. Quantitative image analysis of four angiogenic features (covered area (Blue lines), total tube length, total branching points, and the number of loops) in **(C)** Non-stimulated HUVEC, and TNFα stimulated HUVEC. The analysis was performed by the company WIMASIS. *p < 0.05 compared with the control (no MP). ^#^p < 0.05 respect to Non-Stimulated HUVEC.

The pro-angiogenic potential of MP on non-stimulated and TNFα stimulated HUVEC was determined by measuring four parameters (total tube length, total branching points, total loops, and covered area) (**Figure 8A**) using the tube formation assay. The experiment was performed in both groups of HUVEC after 24h of incubation with and without TNFα and with and without MP. MP enhanced the process of angiogenesis in Non-Stimulated and TNFα Stimulated HUVEC with respect to their control groups (**Figure 8B**). The quantification of the angiogenesis parameters revealed a significant increase in total tube length, total branching points, total loops, and covered area for MP in Non-Stimulated HUVEC, and TNFα Stimulated HUVEC (**Figure 8C**).

## Discussion

The present study demonstrates that small circular fragments of cell membranes from AT-MSC can ameliorate TNFα induced endothelial injury by improving endothelial cell monolayer integrity and enhancing their angiogenic capacity. MP encompass the surface molecules of MSC plasma membranes and contain RNA present in the mother cells, thereby exploiting some of the natural immunomodulatory and regenerative properties of MSC.

The generation of MP as a cell free cell-therapy emerged after our study to heat inactivated MSC (HI-MSC) where we observed that HI-MSC possessed immunomodulatory properties *in vitro and in vivo*, even being dead (14). HI-MSC lost the capacity to secrete factors, or any another function related to the living cells such as proliferation, while keeping the cell membrane intact. This suggests that MSC membranes with their associated proteins can govern at least some of the effects of MSC. The size of HI-MSC is similar as MSC and HI-MSC get trapped in the lung capillary system after their administration (14). To retain the biological properties of MSC and concomitantly overcome the problems of living cells, the generation of MSC membranes in the nano-range devoid of cytoplasm and nucleus represent a new promising approach in the cell therapy field and is supported by the EV studies (22, 23).

MP and EV derived from MSC provide several advantages over MSC. Both types of nanoparticles cannot be modified by the molecular environment after their administration as they are a fixed representation of MSC. Similar to naturally occurring EV, the small size of MP (<200nm) makes them more suitable for crossing the lung barrier than MSC (24, 25) and due to a better biodistribution can exert broadly their effects in the organism (26).

Interestingly, mRNA for factors such as VEGF, IL-8, and CD90 from the MSC were detected in MP. It is assumable that these mRNAs are on the inside of the MP as RNAases would likely degrade free floating RNA. The relative gene expression of these factors in MP samples was different between donors and may be related to the inherent donor variation or to differences in the grade of RNA degradation during the process of MP generation. To minimize the impact on the results due the different amount of mRNA between samples, several batches of MP per donor were used to perform the experiments. One of the mechanisms proposed for explaining the action of EV is the transfer of RNA to the target cells (27, 28). Whether this mechanism is also occurring with MP deserves further studies.

To evaluate whether MP is a potential treatment to repair inflamed endothelium, several aspects of endothelial repair were studied. We showed that MP were efficiently taken up by HUVEC, and that their last destination are the lysosomes of the cells. Recently, we have studied the mechanisms of MP internalization (29). Specific inhibitors for endocytic pathways revealed that MP internalization depends on heparan sulfate proteoglycan-, dynamin-, and clathrin-mediated endocytosis but does not involve caveolin-mediated endocytosis. MP uptake also involved the actin cytoskeleton and phosphoinositide 3-kinase, which are implicated in macropinocytosis and phagocytosis. Due to the different pathways involved in the uptake of MP, the mechanisms involved in their actions may be very different. Several authors described that endocytosis is the most common pathway used by cells to incorporate natural vesicles such as exosomes, and microvesicles to their cytoplasma (30, 31). Bhagyashree S. Joshi et al. (32) demonstrated that EV are internalized by endocytosis and phagocytosis as MP, and the internalized EV fuse with the limiting membrane of endosomes and lysosomes in an acidification-dependent manner, which results in EV cargo exposure to the cell cytosol. MP may be processed by the cells in a similar manner, but future studies should address this question.

Potential adverse effects such as cytotoxicity and upregulation of adhesion molecules on HUVEC by MP were analyzed. No increase in apoptotic HUVEC was observed even with the highest concentration of MP in the inflammatory condition. MP did not have any role on the modulation of the surface adhesion molecules of HUVEC. It is important to highlight that MP did not induce the activation of the HUVEC under normal conditions, which makes them a safe treatment for EC. Because MP did not downregulate the expression of surface adhesion molecules on HUVEC under inflammatory conditions, the adhesion of the monocytes to the activated EC could not be suppressed. Several studies have described the relation of EC adhesion markers and monocyte adhesion. Blocking ICAM-1 receptors (33) in EC, or downregulating the expression of adhesion receptors in EC with molecules such as L-Arginine (34), and Eicosapentaenoic Acid (35) was correlated with a decrease of monocyte adhesion. Although MP did not decrease the number of monocytes adhered to EC, MP were able to prevent the migration of monocytes through a monolayer of HUVEC. Several authors described that MSC inhibit the recruitment of leukocytes (9, 36), but there are some doubts about the mechanisms of action. MSC could physically obstruct the transmigration of leukocytes (37), or immune cells could interact adhesively with MSC thereby reducing the number of cells available to bind to EC (38). MP cannot physically block the migration of monocytes through the barrier of EC, and furthermore in our experiments, MP were removed before the addition of the leukocytes, so they could not interact with the leukocytes themselves. The most likely mechanism explaining the impeding of monocyte transmigration by MP is that MP restore the HUVEC barrier integrity from TNFα-induced leakiness by stimulating a more compact HUVEC monolayer structure. This characteristic of MP is shared with MSC and EV derived from MSC (39, 40).

Additionally, we showed that MP stimulate the angiogenic potential of HUVEC in normal and inflammatory conditions. This effect has also been reported for EV derived from MSC (41), and the described mechanism is through the transfer of miRNAs from EV to the recipient cells (42). It is possible that MP share this mechanism of action with EV as MP also contain mRNAs involved in angiogenesis such as VEGF, angiopoietin 1.

These features of MP, blocking of transmigration, restoring endothelium integrity, and stimulation of angiogenesis could be used in the treatment of different vascular complications such as atherosclerosis, infiltration of immune cells in organ rejection, in the joins in rheumatoid arthritis, or for injured endothelium after organ ischemia. In comparison with similar treatments such as MSC or EV derived from MSC, MP offer the advantage of their small size, purity and excellent safety profile and the possibility for upscaling production in a controlled manner.

In conclusion, MP show a promising medicinal potential, opening a new avenue for treatment of vascular diseases where the inflammatory process is involved in the damage of the endothelium.

## Data Availability Statement

The raw data supporting the conclusions of this article will be made available by the authors, without undue reservation.

## Ethics Statement

The tissues were collected after obtaining written informed consent, as approved by the Medical Ethical Committee of the Erasmus University Medical Centre Rotterdam (protocol no. MEC-2006-190).

## Author Contributions

AM and MH designed the study. AM, SK, and MS performed the research. AM, MH, CL-I, and CB participated in the interpretation of the data. AM wrote the paper. MO-V, CB, EL, and MH wrote the manuscript (review and editing). All authors contributed to the article and approved the submitted version.

## Conflict of Interest

Erasmus MC filed a patent on the use of MP for immunomodulatory purposes. Authors MO-V and EL were employed by Takeda Madrid.

The remaining authors declare that the research was conducted in the absence of any commercial or financial relationships that could be constructed as a potential conflict of interest.
